# eHealth in the Management of Depressive Episodes in Catalonia’s Primary Care From 2017 to 2022: Retrospective Observational Study

**DOI:** 10.2196/52816

**Published:** 2024-01-18

**Authors:** Aïna Fuster-Casanovas, Queralt Miró Catalina, Josep Vidal-Alaball, Anna Escalé-Besa, Carme Carrión

**Affiliations:** 1 eHealth Lab Research Group School of Health Sciences and eHealth Centre Universitat Oberta de Catalunya Barcelona Spain; 2 Unitat de Suport a la Recerca de la Catalunya Central Fundació Institut Universitari per a la Recerca a l'Atenció Primària de Salut Jordi Gol i Gurina Sant Fruitós de Bages Spain; 3 Health Promotion in Rural Areas Research Group Gerència d’Atenció Primària i a la Comunitat de Catalunya Central Institut Català de la Salut Sant Fruitós de Bages Spain; 4 Faculty of Medicine University of Vic-Central University of Catalonia Vic Spain; 5 Network for Research on Chronicity, Primary Care, and Health Promotion Barcelona Spain; 6 School of Medicine Universitat de Girona Girona Spain

**Keywords:** eHealth, depression, depressive disorder, primary health care, mental health patient, patient, patients, healthcare system, digital transformation, mental disorder, mental disorders, diagnostic, clinical practice, clinical practices, retrospective, observational, regression, digital tool, digital tools

## Abstract

**Background:**

The reasons for mental health consultations are becoming increasingly relevant in primary care. The Catalan health care system is undergoing a process of digital transformation, where eHealth is becoming increasingly relevant in routine clinical practice.

**Objective:**

This study aimed to analyze the approach to depressive episodes and the role of eHealth in the Catalan health care system from 2017 to 2022.

**Methods:**

A retrospective observational study was conducted on diagnostic codes related to depressive episodes and mood disorders between 2017 and 2022 using data from the Catalan Institute of Health. The sociodemographic evolution and prevalence of depression and mood disorders in Catalonia were analyzed between 2017 and 2022. Sociodemographic variables were analyzed using absolute frequency and percentage. The prevalence of depressive episodes was calculated, highlighting the year-to-year changes. The use of eHealth for related consultations was assessed by comparing the percentages of eHealth and face-to-face consultations. A comparison of sociodemographic variables based on attendance type was conducted. Additionally, a logistic regression model was used to explore factors influencing face-to-face attendance. The analysis used R software (version 4.2.1), with all differences examined using 95% CIs.

**Results:**

From 2017 to 2022, there was an 86.6% increase in the prevalence of depression and mood disorders, with women consistently more affected (20,950/31,197, 67.2% in 2017 and 22,078/33,169, 66.6% in 2022). In 2022, a significant rise in depression diagnoses was observed in rural areas (difference 0.71%, 95% CI 0.04%-1.43%), contrasting with a significant decrease in urban settings (difference –0.7%, 95% CI –1.35% to –0.05%). There was a significant increase in antidepressant use in 2022 compared to 2017 (difference 2.4%, 95% CI 1.87%-3.06%) and the proportion of eHealth visits rose from 4.34% (1240/28,561) in 2017 to 26.3% (8501/32,267) in 2022. Logistic regression analysis indicated that men (odds ratio [OR] 1.06, 95% CI 1.04-1.09) and younger individuals had a higher likelihood of eHealth consultations in 2022. Furthermore, individuals using eHealth consultations were more likely to use antidepressants (OR 1.54, 95% CI 1.50-1.57) and anxiolytics (OR 1.06, 95% CI 1.03-1.09).

**Conclusions:**

The prevalence of depression in Catalonia has significantly increased in the last 6 years, likely influenced by the COVID-19 pandemic. Despite ongoing digital transformation since 2011, eHealth usage remained limited as of 2017. During the lockdown period, eHealth accounted for nearly half of all health care consultations, representing a quarter of consultations by 2022. In the immediate aftermath of the COVID-19 pandemic, emerging evidence suggests a significant role of eHealth in managing depression-related consultations, along with an apparent likelihood of patients being prescribed antidepressants and anxiolytics. Further research is needed to understand the long-term impact of eHealth on diagnostic practices and medication use.

## Introduction

Depression is a mental disorder that is a major health problem due to its high prevalence, direct repercussions on people's quality of life, and strong social impact [1,2]. According to the World Health Organization, it is estimated that 5% of the world's population suffers from depression [3].

Catalonia, an autonomous community of Spain, had a total of 7,747,709 citizens at the beginning of 2022 [4]. The Catalan health care system, characterized by being public, universal, comprehensive, and equitable, provides health coverage to 7.6 million people [5]. At the level of the Catalan territory, in 2015, according to data from the Catalonia health survey, 16.6% of the population older than 15 years suffered from anxiety or depression, which affected more women (20.8%) than men (12.2%) [6]. In 2020, 5 years later and taking into account the outbreak of the SARS-CoV-2 pandemic, an increase in emotional distress and moderate and major depression was observed. Specifically, 27.19% of women and 17% of men suffered from emotional distress, and 12.2% of women and 5.7% of men had moderate or major depression in Catalonia. Therefore, a decrease in the emotional well-being of the Catalan population has been observed [7].

Primary care (PC), one of the gateways to the health care system, is key in the detection, management, and follow-up of illnesses such as depression. High-quality PC is the foundation of a leading health care system and fundamental to optimizing the performance of the health care system, fulfilling the following 5 dimensions of the quintuple aim: (1) enhancing the care experience, (2) improving population health, (3) reducing costs, (4) care team well-being, and (5) advancing health equity [8-10]. PC has, among other characteristics, longitudinality and universal accessibility that make it the only area designed to be used by all people throughout their lives. As early as 1994, Starfield [11] showed how people living in countries with high-quality PC had better health indices, a more equitable distribution of available resources, and a more efficient health care system.

The reasons for mental health consultations are becoming increasingly relevant in PC, especially among the younger population in the wake of the COVID-19 pandemic [12,13]. Since there is no standardized protocol for mental health care in Catalonia, the approach to these issues varies significantly. This variability could be attributed to differences in professionals’ training or their sensitivity toward handling such problems [14]. Based on the report by the Public Health Agency of Catalonia regarding drug consumption with potential misuse, it was found that antidepressants were the most commonly used group of drugs by the population in 2021. Among most antidepressant drugs, the consumption ratio by women is between 2 to 4 times higher than that by men [15].

Over the last decade, information and communication technology is being introduced into the health care systems of different industrialized countries [16]. Following this trend, the Catalan health care system has been immersed in a digital transformation process. Currently, eHealth within PC in Catalonia involves communication both between providers and patients and amongst providers themselves. These interactions occur via different modes, including telephone or video calls and electronic consultations. It is important to emphasize that although the Catalan health care system had already incorporated technology before, the pandemic served as a clear catalyst for the adoption of eHealth in Catalonia. Regarding the typology of eHealth visits, the majority are conducted via remote consultations (referred to as eConsulta in Catalonia) or phone calls, with video calls being relatively infrequent [17].

Since 2011, several cross-cutting projects of significant importance have been developed in Catalonia, incorporating digital health as a key component [18]. The design of the Catalonia shared medical record began with the aim of being able to share patient information among the different health providers, as well as a personal folder (La Meva Salut) to give citizens access to their personal information, such as current medication plan, diagnoses, vaccines administered, clinical reports, test results, and examinations. Also, within this personal folder, in 2015, eConsulta was developed as an asynchronous digital communication tool involving health care professionals and patients, allowing the population to send queries at any time to their PC doctor or nurse and receive a response within a maximum of 48 hours on working days [19,20]. In this sense, eConsulta represents a significant advancement in eHealth within the Catalan public health system. Prior to the pandemic, eConsulta usage had a monthly growth rate of 7%; from March 15 to May 2020, an exponential growth rate was observed [21]. A study focusing on the profile of health care professionals who utilized remote consultations like eConsulta before the pandemic revealed that physicians engaging with eConsulta were typically aged between 45 and 64 years, exceeded the 80th percentile in the Quality of Care Index, had a high level of accessibility to their patients, participated in educational activities, and operated within a health team framework in urban settings with a high socioeconomic status [22].

In the context of Catalan PC, eHealth has been widely implemented and adopted in routine clinical practice, even more so in the context of COVID-19 [23-25]. However, there are other digital health tools with great potential, such as mobile health or artificial intelligence, that have not yet been introduced into the public health care system in Catalonia.

In order to improve the management of depressive episodes in PC in the Catalan health care system and to obtain higher effectiveness rates close to the potential efficacy of the available treatments, the aim of this study was to analyze the evolution of the prevalence of depression and mood disorders in PC from 2017 to 2022, to examine the sociodemographic profile of the affected population, and to investigate the role played by eHealth in this context and assess its impact on consultations related to depression and mood disorders.

## Methods

### Study Design

This study was a retrospective observational study of diagnostic codes related to depressive episodes and mood disorders between 2017 to 2022 from the Catalan Institute of Health.

### Sample

We analyzed the entire population of Catalonia that visited PC centers of the Catalan Institute of Health who had a face-to-face or eHealth consultations (eConsulta, telephone, or video consultations) associated with the selected diagnoses of depression and mood disorders in the period of January 2017 to December 2022. The Catalan Institute of Health is the main provider of health services in the public system. It manages approximately 80% of the PC teams in Catalonia and provides health care coverage to approximately 5.8 million people [16,21,26,27].

To obtain the study sample, the diagnostic codes were selected according to those typified in the International Statistical Classification of Diseases and Related Health Problems (ICD-10), which is the classification used by PC professionals when recording a diagnosis in the computerized PC clinic [28]. All diagnostic codes related to depression (mild, moderate, and major) and mood disorders (eg, cyclothymia and dysthymia) that are usually detected, managed, or followed up in Catalan PC were selected (Table S1 in Multimedia Appendix 1). Mood disorders are marked by persistent clinical manifestations. In the case of dysthymia, it necessitates at least 2 years of a consistently low mood, whereas cyclothymia involves oscillations between a depressed mood and euphoria, without fulfilling the criteria for major depression [29]. Therefore, these diagnoses were included because they are not reactive disorders of short duration. By including them, there is a reduced risk of missing cases of mild depressive disorders.

Finally, we excluded all consultations that did not have an associated diagnosis code among those selected. The part of the population of Catalonia whose public health coverage was not provided by the Catalan Institute of Health was also excluded.

The database was obtained through the Information System for the Development of Research in Primary Care [30].

### Variables

To study sociodemographic characteristics, we considered age, sex, drug use, recurrent depressive episode diagnoses, rurality, and the socioeconomic situation recorded through the MedeA index [31]. A variable was created to distinguish between individuals with recurrent depressive episode diagnoses (where depression had been diagnosed previously in their medical history) and those without such recurrent diagnoses. The MedeA is a deprivation index linked to each census tract of the population. This assessment focuses on the barriers to access employment, education, culture, and social development. The aim is to evaluate them at a level that is considered acceptable within the surrounding society or region. The assessment comprises subindicators for employment and education. It is only available for urban areas, which are defined as municipalities with more than 10,000 inhabitants and a population density of more than 150 inhabitants per km^2^. Other areas were considered rural. The MedeA index is ranked in quintiles, from MedeA urban 1 indicating low deprivation to MedeA urban 5 indicating high deprivation [31]. The MedeA index was used to categorize rurality by grouping urban and rural groups.

### Statistical Analysis

To observe the evolution of the profile of people diagnosed with depression and mood disorders, sociodemographic variables were described in 2017 and 2022. Categorical variables were described by absolute frequency and percentage. The difference between the years was calculated with percentage points.

To observe the evolution of the prevalence of depressive episodes and mood disorders over the years for the total sample, the prevalent cases for each year were divided with respect to the total population assigned to the Catalan Institute of Health PC centers throughout Catalonia. The percentage change was calculated to determine the year-to-year evolution of these prevalences.

To observe the use of eHealth in consultations related to depression and mood disorders over the years, the percentage of telematic and face-to-face consultations resulting in new diagnoses per year was calculated.

A comparison of the sociodemographic variables as a function of face-to-face attendance was performed. Categorical variables were described by absolute frequency and percentage. The difference was calculated as percentage points.

Lastly, a logistic regression model was applied to observe how the studied variables affected face-to-face attendance. The model incorporated the variables of sex, age, rurality, recurrence, antidepressants, and anxiolytics. The MedeA variable was not introduced into this model since the rurality variable was used.

Analyses were performed with R version 4.2.1 (R Foundation for Statistical Computing). All differences were examined using CIs, and a confidence level of 95% was established.

### Ethics Approval

The study protocol was approved by the University Institute for Primary Care Research (IDIAP) Jordi Gol Health Care Ethics Committee (Code 23/013-P).

## Results

### Description of Sociodemographic Profiles of New Cases From 2017 to 2022

An analysis of the sociodemographic profile of new cases during 2017 (n=31,197) and 2022 (n=33,169) was performed to observe changes in the characteristics of this population group during the study period (Table 1).

**Table 1:** Comparison of demographic characteristics of new cases in 2017 and 2022.

		2017 (n=31,197), n (%)	2022 (n=33,169), n (%)	Absolute difference (%) from 2017 to 2022 (95% CI)
**Age (years)**				
	0-15	689 (2.21)	922 (2.78)	0.57 (0.33 to 0.81)^a^
	16-24	1437 (4.61)	2415 (7.28)	2.67 (2.31 to 3.04)^a^
	25-34	2727 (8.74)	3712 (11.2)	2.46 (1.98 to 2.91)^a^
	35-44	4924 (15.8)	4765 (14.4)	–1.4 (–1.97 to –0.86)
	45-54	5653 (18.1)	6101 (18.4)	0.3 (–0.32 to 0.87)
	55-64	5355 (17.2)	5754 (17.3)	0.1 (–0.41 to 0.76)
	65-74	4094 (13.1)	3860 (11.6)	–1.5 (–1.99 to –0.87)^a^
	75-84	4171 (13.4)	3670 (11.1)	–2.3 (–2.81 to –1.79)^a^
	≥85	2147 (6.88)	1970 (5.94)	–0.94 (–1.32 to –0.56)^a^
**Gender**				
	Women	20,950 (67.2)	22,078 (66.6)	–0.6 (–1.30 to 0.15)
	Men	10,222 (32.8)	11,057 (33.4)	—^b^
**MedeA index**				
	Rural	7312 (25.6)	8499 (26.33)	0.71 (0.04 to 1.43)^a^
	Urban 1	6036 (21.1)	6592 (20.4)	–0.7 (–1.35 to –0.05)^a^
	Urban 2	4269 (14.94)	4806 (14.9)	0 (–0.62 to 0.51)
	Urban 3	5644 (19.76)	6419 (19.9)	0.1 (–0.50 to 0.77)
	Urban 4	5300 (18.55)	5951 (18.4)	–0.2 (–0.73 to 0.50)
**Recurrent**				
	No	29,144 (93.4)	29,967 (90.3)	–3.07 (–3.49 to –2.65)^a^
	Yes	2053 (6.58)	3202 (9.65)	3.07 (2.65 to 3.49)^a^
**Face-to-face**				
	eHealth	1240 (4.34)	8501 (26.3)	22 (21.46 to 22.54)^a^
	Face-to-face	27,321 (95.7)	23,766 (73.7)	—
**Rurality**				
	Rural	7312 (25.6)	8499 (26.3)	0.7 (0.04 to 1.43)^a^
	Urban	21,249 (74.4)	23,768 (73.7)	—
**Antidepressants**				
	Yes	25,353 (81.3)	27,777 (83.7)	2.4 (1.87 to 3.06)^a^
	No	5844 (18.7)	5392 (16.3)	—
**Anxiolytics**				
	Yes	15,615 (50.1)	16,519 (49.8)	–0.3 (–1.02 to 052)
	No	15,582 (49.9)	16,650 (50.2)	—

^a^Statistically significant difference.

^b^Given the symmetry of the binary variables, the difference and 95% CI have only been expressed for one of the categories.

The results showed that in both 2017 and 2022, the 45-54 years age range had the most diagnoses related to depressive episodes and mood disorders. However, in 2022 there was a significant increase compared to 2017 in diagnoses in the younger age ranges (0-15 years: difference 0.57%, 95% CI 0.33%-0.81%; 16-24 years: difference 2.67%, 95% CI 2.31%-3.04%; 25-34 years: difference 2.46%, 95% CI 1.98%-2.91%). A significant reduction in diagnoses was also observed in the older age ranges (65-74 years: difference –1.5%, 95% CI –1.99% to –0.87%; 75-84 years: difference –2.3%, 95% CI –2.81 to –1.79) and among those older than 85 years (difference –0.94%, 95% CI –1.32% to –0.56%).

Regarding gender, it was observed that in both 2017 and 2022, women were the most affected gender (20,950/31,172, 67.2% and 22,078/33,135, 66.6%, respectively). Regarding the MedeA index, a significant increase in diagnoses was observed in rural settings in 2022 with respect to 2017 (difference 0.71%, 95% CI 0.04%-1.43%), while a significant decrease was observed in urban settings, specifically in the urban population with low deprivation (difference –0.7%, 95% CI –1.35% to –0.05%). Regarding recurrent depressive episodes, there was a pronounced and significant increase in those diagnosed as recurrent compared to those diagnosed with a depressive episode for the first time (difference 3.07%, 95% CI 2.65%-3.49%). Regarding the use of eHealth for managing these illnesses, a notable shift was observed in the pattern of face-to-face attendance for the management of depressive episodes and mood disorders. A significant increase was observed in the use of eHealth as the approach for these diagnostic codes (difference 22%, 95% CI 21.46%-22.54%). Finally, regarding the most common medication used for depressive episodes and mood disorders, a significant increase in the use of antidepressants was observed in 2022 compared to 2017 (difference 2.4%, 95% CI 1.87%-3.06%), while no differences were observed in the use of anxiolytics.

### Evolution of the Prevalence of Depressive Episodes and Mood Disorders by Year

**Figure 1:**
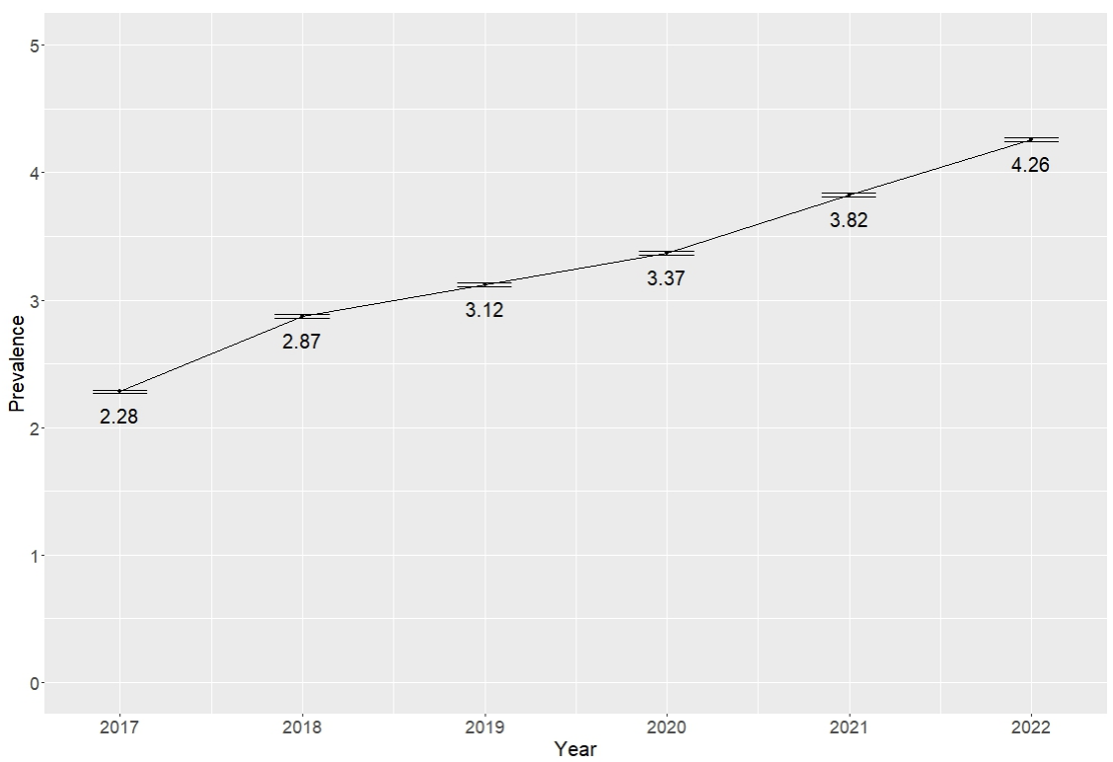
Depression prevalence (%) with 95% CIs between years.

The prevalence per year of all selected diagnostic codes was calculated during the period from 2017 to 2022, considering the total population assigned to the Catalan Institute of Health Primary Care Centers throughout Catalonia (Figure 1). Since 2017, an increase in diagnoses was observed. The steepest increase was in 2018, which went from 2.3% (95% CI 2.27%-2.29%) during 2017 to 2.9% (95% CI 2.86%-2.88%) in 2018. From 2019 to 2022, a progressive increase in prevalence was observed, reaching 4.3% (95% CI 4.24%-4.27%) in 2022. In short, the prevalence of depressive episodes and mood disorders increased by 86.6% during the study period. The results can be seen in Table S2 in Multimedia Appendix 1.

### Use of eHealth for Consultations Related to Depression and Mood Disorders by Year

**Figure 2:**
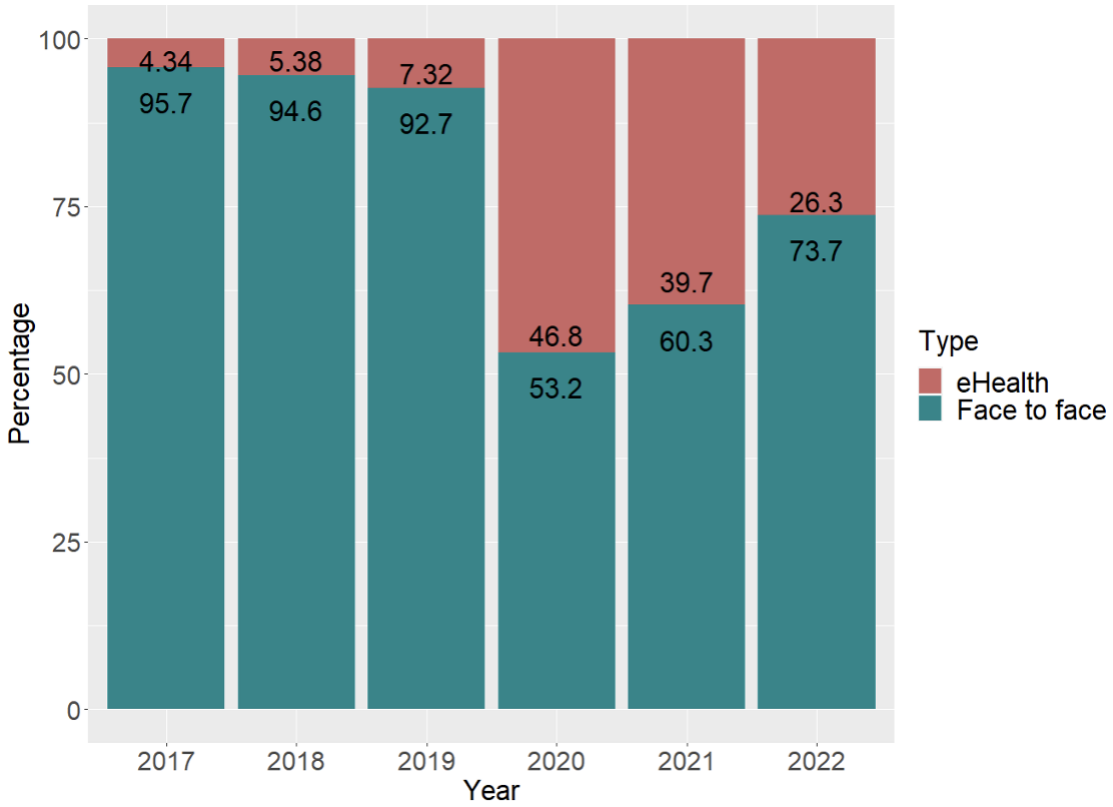
Percentage of telematic and face-to-face consultations from 2017 to 2022.

The percentage of face-to-face and eHealth visits for each year of new diagnoses was calculated to analyze the use of eHealth for consultations related to depression and mood disorders in the public health care system (Figure 2). It was noted that in 2017 eHealth consultations represented 4.34% (1240/28,561) of all depressive episode and mood disorder visits, with an increase during 2018 and 2019. As of 2020, coinciding with the pandemic lockdown, eHealth consultations accounted for 46.8% (11,122/23,790), representing a more than 6-fold increase over the previous year. In 2021, there was some recovery of face-to-face consultations, but nevertheless, eHealth consultations still accounted for 39.7% (12,119/30,561) of total consultations. Finally, in 2022, eHealth consultations accounted for 26.3% (8501/32,267), representing just over a quarter of all consultations for diagnoses of depression and mood disorders.

### Comparison of Prevalent Cases in 2022 According to Face-to-Face Attendance

A comparative analysis of the sociodemographic variables of the prevalent 2022 cases was performed according to face-to-face consultations (Table 2). Regarding age, it was observed that the populations that used eHealth consultations the most were those aged 0 to 15 years (difference –1.18%, 95% CI –1.36% to –0.98%), 16 to 24 years (difference –0.64%, 95% CI –0.89% to 0.38%), and those aged 85 years and older (difference –4.37%, 95% CI –4.68% to –4.05). In contrast, in 2022, it was observed that the populations that used face-to-face consultations the most were people aged 35 to 44 years (difference 0.7%, 95% CI 0.33%-1.10%), 45 to 54 years (difference 0.7%, 95% CI 0.27%-1.11%), 55 to 64 years (difference 0.8%, 95% CI 0.43%-1.24%), 65 to 74 years (difference 3.1%, 95% CI 2.84%-3.53%), and 75 to 84 years (difference 0.59%, 95% CI 0.59%-1.31%).

Significant differences were also observed in the MedeA index. People living in urban settings with less deprivation (urban 1) made more use of eHealth (difference –1.9%, 95% CI –2.35% to –1.44%), while those with higher deprivation in urban settings (urban 3 and urban 4) used face-to-face consultations more often (difference 0.9%, 95% CI 0.48%-1.34% and difference 2.4%, 95% CI 2.01%-2.84%, respectively). Regarding recurrence, it was observed that individuals with recurrent depressive episodes sought consultation more frequently via eHealth (difference –2.46%, 95% CI –2.77% to –2.15%). In terms of rurality, individuals living in rural areas demonstrated a higher frequency of eHealth usage (difference –1.4%, 95% CI –1.94% to –0.97%). Finally, individuals receiving treatment with antidepressants or anxiolytics showed a higher tendency to use eHealth services for their health consultations (difference –10.1%, 95% CI –10.59% to –9.50% and difference –4%, 95% CI –4.53% to –3.51%, respectively).

**Table 2:** Comparative table of prevalent cases in 2022 according to attendance type.

			Non–face-to-face (n=38,719), n (%)		Face-to-face (n=188,598), n (%)	Absolute difference (%) between attendance types (95% CI)
**Age** **(years)**						
	0-15	1246 (3.22)		3850 (2.04)		–1.18 (–1.36 to –0.98)^a^
	16-24	2219 (5.73)		9599 (5.09)		–0.64 (–0.89 to –0.38)^a^
	25-34	3615 (9.34)		17,240 (9.14)		–0.2 (–0.51 to 0.12)
	35-44	5616 (14.5)		28,711 (15.2)		0.7 (0.33 to 1.10)^a^
	45-54	6859 (17.7)		34,716 (18.4)		0.7 (0.27 to 1.11)^a^
	55-64	6388 (16.5)		32,688 (17.3)		0.8 (0.43 to 1.24)^a^
	65-74	4086 (10.6)		25,913 (13.7)		3.1 (2.84 to 3.53)^a^
	75-84	4742 (12.2)		24,894 (13.2)		1 (0.59 to 1.31)^a^
	≥85	3948 (10.2)		10,987 (5.83)		–4.37 (–4.69 to –4.05)^a^
**Gender**						
	Women	25,802 (66.7)		126,652 (67.2)		0.5 (0.00 to 1.03)
	Men	12,886 (33.3)		61,832 (32.8)		—^b^
**MedeA** **index**						
	Rural	10,418 (26.9)		48,003 (25.5)		–1.4 (–1.94 to –0.97)^a^
	Urban 1	8687 (22.4)		38,726 (20.5)		–1.9 (–2.35 to –1.44)^a^
	Urban 2	5838 (15.1)		28,469 (15.1)		0 (–0.37 to 0.41)
	Urban 3	7363 (19)		37,588 (19.9)		0.9 (0.48 to 1.34)^a^
	Urban 4	6413 (16.6)		35,812 (19)		2.4 (2.01 to 2.84)^a^
**Recurrent**						
	No	35,209 (90.9)		176,141 (93.39)		—
	Yes	3510 (9.07)		12,457 (6.61)		–2.46 (–2.77 to –2.15)^a^
**Rurality**						
	Rural	10,418 (26.9)		48,003 (25.5)		–1.4 (–1.94 to –0.97)^a^
	Urban	28,301 (73.1)		140,595 (74.5)		—
**Antidepressants**						
	Yes	20,464 (52.9)		80,734 (42.8)		–10.1 (–10.59 to –9.50)^a^
	No	18,255 (47.1)		107,864 (57.2)		—
**Anxiolytics**						
	Yes	12,819 (33.1)		54,855 (29.1)		–4 (–4.53 to –3.51)^a^
	No	25,900 (66.9)		133,743 (70.9)		—

^a^Statistically significant differences.

^b^Given the symmetry of the binary variables, the difference and CI have only been expressed for one of the categories.

**Figure 3:**
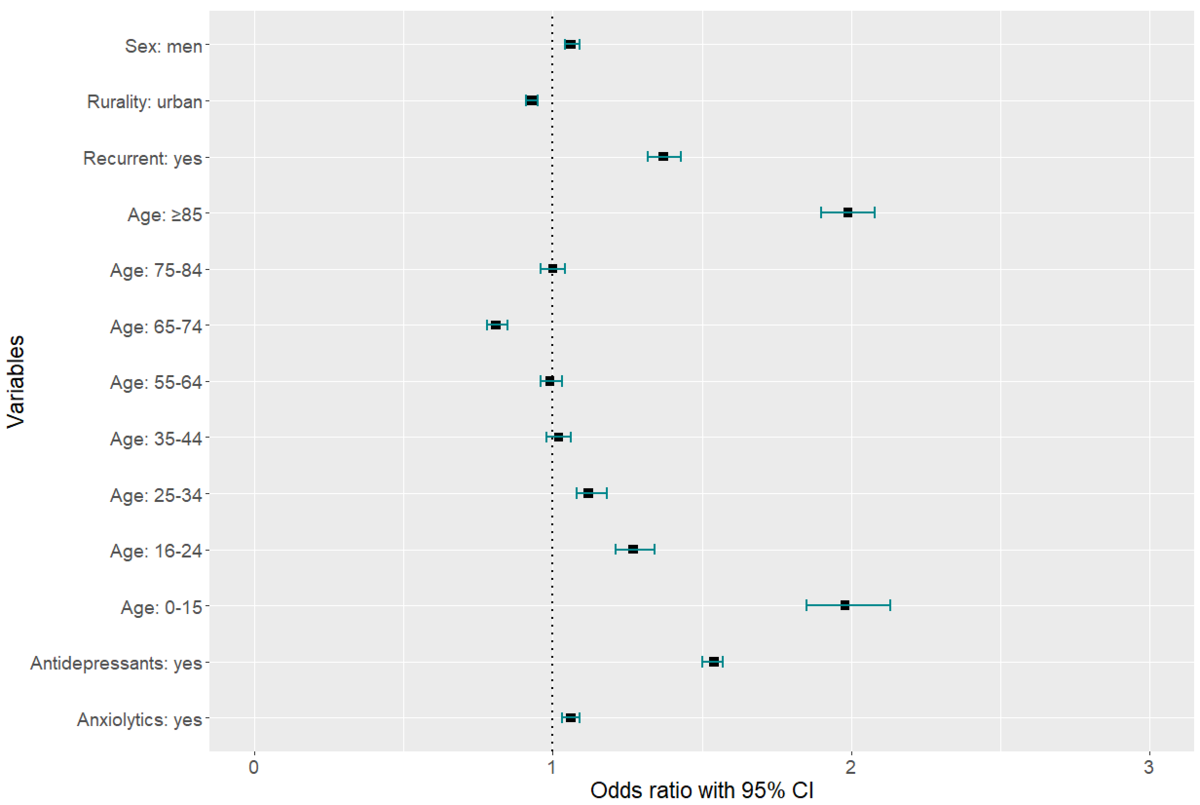
Logistic regression results. The reference categories were as follows: women, aged 45-54, nonrecurrent, rural, no antidepressants, and no anxiolytics. The black squares indicate the odds ratio estimate, and the horizontal lines indicate the 95% CI.

Finally, a logistic regression model was used to observe the impact of the studied variables from the 2022 attendance data (Figure 3). Men were 1.06 times more likely (95% CI 1.04-1.09) to use eHealth in consultations than women, and those suffering from recurrent depressive episodes were 1.37 times more likely (95% CI 1.32-1.43) to use eHealth in consultations. It was also observed that the youngest age ranges (0-15 years: odds ratio [OR] 1.98, 95% CI 1.85-2.13; 16-24 years: OR 1.27, 95% CI 1.21-1.34; 25-34 years: OR 1.12, 95% CI 1.08-1.18) were more likely to use eHealth in consultations, as was also the case among those aged 85 and older (OR 1.99, 95% CI 1.90-2.08). In contrast, people living in urban settings were 7% less likely (OR 0.93, 95% CI 0.91-0.95) to have an eHealth consultation. Finally, people who consulted more through eHealth were 1.54 times (95% CI 1.50-1.57) more likely to take an antidepressant and 1.06 times (95% CI 1.03-1.09) more likely to take an anxiolytic. The results can be seen in Table S3 in Multimedia Appendix 1.

## Discussion

### Principal Findings

The aim of the present study was to analyze the context of depression within the Catalan health care system. Consequently, the prevalence of depression and mood disorders has been successfully analyzed over the years, along with the sociodemographic profile of this population. Additionally, the role played by eHealth and its impact on PC for these conditions has been studied.

Regarding the prevalence of depression and mood disorders in Catalan PC, an increase of 86.6% was observed during the study period. One possible reason explaining this increase in prevalence could be an uptick in the detection of these cases within the public health care system, possibly indicating increased awareness among both professionals and citizens. In 2006 in Catalonia, the Primary Care Support Programme was implemented to coordinate PC with mental health services, providing a budget to hire more psychiatrists and psychologists in order to reinforce PC [32,33]. However, it was not until 2017 that implementation was completed in all PC centers in Catalonia. In this context, despite the existence of critical reports regarding the operation of this support program, its initiation might have positively influenced professionals’ awareness, potentially contributing to an increase in detections in PC since then. The results of the study revealed a more pronounced increase (25.82%) in 2018, coinciding with the end of this program’s operation.

Another possible reason explaining the increase in prevalence is the COVID-19 pandemic. The data analyzed cover the period from before the pandemic to the early postpandemic period, so the results may indeed be influenced by the consequences of lockdown and the isolation of individuals. In this context, the pandemic has generated great concern and an even more marked increase in mental health awareness, especially among the younger population. As the context of social isolation has strongly impacted the mental health of young people, as it is a crucial period for the development of social skills, it is noteworthy that from the prepandemic context in 2019, the overall prevalence increased by 36.52% in 2022 [34,35]. However, in line with the statistics already published, it is worth mentioning that women are the most affected group [6,7]. Over the past decade, the importance of sex- and gender-stratified biomedical research has grown significantly, as demonstrated by the observations of Oertelt-Prigione et al [36] of an increasing number of publications in medicine addressing this issue. It is now crucial to incorporate this perspective into the clinical approach to depression. Understanding how individuals’ behaviors align with social expectations, coping strategies, and help-seeking tendencies can contribute to a more comprehensive approach to treatment. Regarding the medicalization of these conditions, it has been observed that approximately 80% of new diagnoses of depression and mood disorders in both 2017 and 2022 were prescribed antidepressant drugs. That is, most people who were diagnosed for the first time were associated with antidepressants. In contrast, the prevalence of antidepressant use has been found to be around 40% to 50% in the entire prevalent population. The results suggest that although most people who are diagnosed with depression or mood disorders are prescribed medication, in most cases treatment is withdrawn, as indicated by clinical practice guidelines [37,38].

Regarding the use of eHealth for consultations on depression and mood disorders in the PC context in Catalonia, it has been observed that the use of eHealth is increasingly being integrated into daily clinical practices in Catalonia [18]. In fact, while in 2017 diagnoses made through eHealth consultations accounted for only 4.34% of consultations, in 2022 they accounted for 26.34%. Without a doubt, the pandemic has also been a clear catalyst in the use of eHealth. Although the volume of eHealth consultations in 2020 was not equivalent to that in 2022, the results from the latter year may suggest a shift in the pattern of consultations related to depression and mood disorders. In this sense, eHealth is gaining prominence in routine practice, as has been suggested by other studies conducted in Catalonia [17,39,40]. Nevertheless, the incorporation of eHealth into the health care system must be accompanied by training for the professionals who must use it. In other words, it is not just about providing infrastructure and resources for technology operation. It is also crucial to understand when the use of these tools aligns with the care process and equip the health care professional to make clinical and therapeutic decisions appropriately, as they hold responsibility for the procedure and its outcomes [41]. The results of the present study have also shown that one of the impacts of eHealth on depressive episodes and mood disorders is on the prescription of drugs. People treated with eHealth were prescribed 54% more antidepressant drugs and 6% more anxiolytics. A systematic review by Han et al [42] observed the impact of eHealth on antibiotic prescribing in PC. Although it was not possible to conclude with sufficient confidence that eHealth significantly impacts the prescribing of these drugs, 4 of the 12 studies analyzed reported higher prescribing rates through eHealth [42]. In a study conducted by Wabe et al [43] during the pandemic in Australia's PC, they compared the prescription of medication through face-to-face consultation versus eHealth methods. The findings revealed that prescribing through face-to-face consultations was more prevalent for all drug groups classified in the World Health Organization Anatomical Therapeutic Chemical Classification list [44], except for the group that includes nervous system drugs, such as antidepressants and anxiolytics. In the context of PC in Catalonia, it would be interesting to examine whether the results obtained related to the increased prescription of drugs via eHealth can be applied to other medical conditions. In this regard, the development strategies used in the approach to eHealth will be crucial to ensure consistency in clinical practice.

### Strengths and Limitations

The most relevant strength of this study is the size of the sample, since it was possible to work with the consultations related to depressive episodes and mood disorders of almost 5.8 million people. As a result, this study has yielded robust and realistic findings regarding the prevalence of depression, the use of eHealth, and the associated implications. Another strength of this study lies in the comprehensive analysis of the role and implications of eHealth in depression-related consultations. It not only highlights the current utilization of eHealth in depression consultations but also underscores the need to develop strategies that promote consistency between in-person and eHealth visits.

The main limitation of this analysis is that the data were collected only from the Catalan Institute of Health within Catalonia’s public health care system. As a result, there is a lack of information from individuals who have sought treatment through private health care providers. Another limitation is that, since this was a cross-sectional study, it was not possible to follow up on each individual user, and therefore, it was not possible to establish a causal relationship between the variables studied, although the relationship between them was observed. However, due to the absence of subdivision in the original database regarding telematic consultations, specifically whether they were synchronous or asynchronous, a detailed breakdown of the proportions of telematic consultations could not be provided. Furthermore, it is essential to acknowledge the limitation that the data analyzed may reflect the impact of the lockdown imposed during de COVID-19 pandemic. The pandemic led to the isolation of individuals, which, in turn, resulted in a notable increase in diagnoses through eHealth and prescriptions of antidepressants and anxiolytics when comparing the period before COVID-19 to the postlockdown situation. In this context, the often-mandatory use of eHealth consultations during the lockdown, along with the isolation of the population, may have contributed to the increase in the detection rates and medication usage reflected in the results. Therefore, it would be valuable to replicate this study in the coming years to reassess the prevalence and sociodemographic characteristics of depression as well as the role of eHealth in addressing the observed diagnostic and pharmacological trends. This follow-up study would enable us to determine whether the findings observed in this study represent short-term consequences of COVID-19 or if they have more lasting implications over time.

### Conclusions

The prevalence of depression in Catalonia has significantly increased from 2017 to 2022, with the pandemic likely having a profound impact. Although the Catalan health care system has been undergoing digital transformation since 2011, eHealth usage was limited in 2017. During the lockdown, it accounted for nearly half of the care, and by 2022 it represented a quarter of the consultations. In the short post-COVID-19 era, evidence suggests that eHealth plays a role in consultations related to depression and appears to increase the likelihood of taking antidepressants and anxiolytics. However, future studies in a context further removed from the pandemic could provide a more accurate indication of the role of eHealth in diagnosing and treating depression and mood disorders.

## Appendix

Multimedia Appendix 1 Selected diagnostic codes, depression prevalence, and logistic regression.

## Abbreviations

ICD-10: International Statistical Classification of Diseases and Related Health Problems
OR: odds ratio
PC: primary care

## Data Availability

The data sets generated and analyzed during the current study are not publicly available because the manuscript was based on confidential and sensitive health data but are available from the corresponding author upon reasonable request.

## References

[1] (2021). Depression. World Health Organization.

[2] (2022). Depressió. Generalitat de Catalunya.

[3] (2021). Depressive disorder (depression). World Health Organization.

[4] (2022). Població. Generalitat de Catalunya.

[5] (1990). LLEI 15/1990, de 9 de juliol, d'ordenació sanitària de Catalunya. Generalitat de Catalunya.

[6] Garcia O, Medina A, Schiaffino A Comportaments relacionats amb la salut, l’estat de salut i l’ús de serveis sanitaris a Catalunya: informe dels principals resultats 2015. Generalitat de Catalunya.

[7] L’estat de salut, els comportaments relacionats amb la salut i l’ús de serveis sanitaris a Catalunya 2021. Generalitat de Catalunya.

[8] (2022). Triple aim and population health. Institute for Healthcare Improvement.

[9] Nundy S, Cooper LA, Mate KS (2022). The quintuple aim for health care improvement: a new imperative to advance health equity. JAMA.

[10] Bodenheimer T, Sinsky C (2014). From triple to quadruple aim: care of the patient requires care of the provider. Ann Fam Med.

[11] Starfield B (1994). Is primary care essential?. Lancet.

[12] (2020). La COVID-19 y la necesidad de actuar en relación con la salud mental. Naciones Unidas.

[13] (2021). Efectes del confinament en la salut mental (42/2021). Generalitat de Catalunya.

[14] Fernández de Sanmamed J, González Y, Mazo V, Pons G, Robles I, Serrano E, Teixido A, Zapater F (2020). Al voltant del model d'atenció a la salut mental a l'atenció primària de salut. Fòrum Català d'Atenció Primària.

[15] (2021). Informe sobre consum problemàtic i conseqüències: consum de fàrmacs amb potencial d'abús. Generalitat de Catalunya.

[16] Baltaxe E, Cano I, Herranz C, Barberan-Garcia A, Hernandez C, Alonso A, Arguis MJ, Bescos C, Burgos F, Cleries M, Contel JC, de Batlle J, Islam K, Kaye R, Lahr M, Martinez-Palli G, Miralles F, Moharra M, Monterde D, Piera J, Ríos J, Rodriguez N, Ron R, Rutten-van Mölken M, Salas T, Santaeugenia S, Schonenberg H, Solans O, Torres G, Vargiu E, Vela E, Roca J (2019). Evaluation of integrated care services in Catalonia: population-based and service-based real-life deployment protocols. BMC Health Serv Res.

[17] Lopez Segui F, Hernandez Guillamet G, Pifarré Arolas H, Marin-Gomez FX, Ruiz Comellas A, Ramirez Morros AM, Adroher Mas C, Vidal-Alaball J (2021). Characterization and identification of variations in types of primary care visits before and during the COVID-19 pandemic in Catalonia: big data analysis study. J Med Internet Res.

[18] Plade salut de Catalunya 2011-2015. Generalitat de Catalunya.

[19] (2022). La meva salut. Generalitat de Catalunya.

[20] (2021). eConsulta. Generalitat de Catalunya.

[21] López Seguí F, Walsh S, Solans O, Adroher Mas C, Ferraro G, García-Altés A, García Cuyàs F, Salvador Carulla L, Sagarra Castro M, Vidal-Alaball J (2020). Teleconsultation between patients and health care professionals in the Catalan primary care service: message annotation analysis in a retrospective cross-sectional study. J Med Internet Res.

[22] Fernández OS, Seguí FL, Vidal-Alaball J, Bonet Simo JM, Vian OH, Cabo PR, Hernandez MC, Dominguez CO, Reig XA, Rodríguez YD, Peralta MM, Hermosilla E, León NM, Guimferrer N, González MA, Cuyàs FG, Sust PP (2020). Primary care doctor characteristics that determine the use of teleconsultations in the Catalan public health system: retrospective descriptive cross-sectional study. JMIR Med Inform.

[23] Solans O, Vidal-Alaball J, Roig Cabo P, Mora N, Coma E, Bonet Simó JM, Hermosilla Pérez E, Saigí-Rubió F, Olmos Domínguez C, Piera-Jiménez J, Abizanda González M, López Seguí F (2021). Characteristics of citizens and their use of teleconsultations in primary care in the Catalan public health system before and during the COVID-19 pandemic: retrospective descriptive cross-sectional study. J Med Internet Res.

[24] Vidal-Alaball J, López Seguí F (2020). Ha llegado para quedarse: beneficios e inconvenientes de la eConsulta. Atención Primaria Práctica.

[25] López Seguí F, Vidal-Alaball J, Sagarra Castro M, García-Altés A, García Cuyàs F (2020). General practitioners' perceptions of whether teleconsultations reduce the number of face-to-face visits in the Catalan public primary care system: retrospective cross-sectional study. J Med Internet Res.

[26] Institut CDLS (2021). Institut Català de la Salut memòria 2020. Generalitat de Catalunya.

[27] Entitats proveïdores d'atenció primària. Generalitat de Catalunya.

[28] (2022). CIM-10-MC/SCP. Generalitat de Catalunya.

[29] Markon KE, Fossati A, Somma A, Krueger B (2014). Guía de Consulta de los Criterios Diagnósticos del DSM-5.

[30] SIDIAP.

[31] Domínguez-Berjón MF, Borrell C, Cano-Serral G, Esnaola S, Nolasco A, Pasarín MI, Ramis R, Saurina C, Escolar-Pujolar A (2008). Constructing a deprivation index based on census data in large Spanish cities (the MEDEA project). Gac Sanit.

[32] (2017). Pla director de salut mental i addiccions. Generalitat de Catalunya.

[33] (2021). Consideracions sobre l'atenció a la salut mental en l'àmbit sanitari d'Atenció Primària. Metges de Catalunya.

[34] (2020). Salud mental e infancia en el escenario de la COVID-19. Propuestas de UNICEF España. UNICEF España.

[35] (2021). Pla de salut 2021-2025. Generalitat de Catalunya.

[36] Oertelt-Prigione S, Parol R, Krohn S, Preissner R, Regitz-Zagrosek V (2010). Analysis of sex and gender-specific research reveals a common increase in publications and marked differences between disciplines. BMC Med.

[37] (2022). Depression in adults: treatment and management NICE guideline. National Institute for Health and Care Excellence.

[38] (2014). Guía de práctica clínica sobre el manejo de la depresión en el adulto. Ministerio de Sanidad, Servicios Sociales e Igualdad.

[39] Coma E, Miró Q, Medina M, Marin-Gomez FX, Cos X, Benítez M, Mas A, Fàbregas M, Fina F, Lejardi Y, Vidal-Alaball J (2021). Association between the reduction of face-to-face appointments and the control of patients with type 2 diabetes mellitus during the Covid-19 pandemic in Catalonia. Diabetes Res Clin Pract.

[40] López Seguí F, Ander Egg Aguilar R, de Maeztu G, García-Altés A, García Cuyàs F, Walsh S, Sagarra Castro M, Vidal-Alaball J (2020). Teleconsultations between patients and healthcare professionals in primary care in Catalonia: the evaluation of text classification algorithms using supervised machine learning. Int J Environ Res Public Health.

[41] (2021). Telemedicina: com i quan utilitzar-la en la pràctica assistencial. Quad la Bona Praxi.

[42] Han SM, Greenfield G, Majeed A, Hayhoe B (2020). Impact of remote consultations on antibiotic prescribing in primary health care: systematic review. J Med Internet Res.

[43] Wabe N, Thomas J, Sezgin G, Sheikh MK, Gault E, Georgiou A (2021). Medication prescribing in face-to-face versus telehealth consultations during the COVID-19 pandemic in Australian general practice: a retrospective observational study. BJGP Open.

[44] Anatomical therapeutic chemical (ATC) classification. World Health Organization.

