# Supplementary material for: eHealth in the Management of Depressive Episodes in Catalonia’s Primary Care From 2017 to 2022: Retrospective Observational Study
Source: JMIR Ment Health. 2024 Jan 18;11:e52816. doi: 10.2196/52816 (PMC10835588; doi:10.2196/52816)
Supplement: Multimedia Appendix 1 [file mental_v11i1e52816_app1.docx]

## Appendix I. Selected diagnostic codes

Table S1. Diagnostic codes

| diagnostic code | description |
| --- | --- |
| F32.0 | Mild depressive episode |
| F32.1 | Moderate depressive episode |
| F32.2 | Major depressive episode without psychotic symptoms |
| F32.3 | Major depressive episode with psychotic symptoms |
| F32.8 | Another depressive episode |
| F32.9 | Unspecified depressive episode |
| F33.0 | Recurrent depressive disorder, current mild episode |
| F33.1 | Recurrent depressive disorder, current moderate episode |
| F33.2 | Recurrent depressive disorder, current major episode without psychotic symptoms |
| F33.3 | Recurrent depressive disorder, current major episode with psychotic symptoms |
| F33.4 | Recurrent depressive disorder, currently in remission |
| F33.8 | Another recurrent depressive episode |
| F33.9 | Unspecified recurrent depressive episode |
| F34.0 | Cyclothymia |
| F34.1 | Dysthymia |
| F34.8 | Other persistent mood disorders |
| F34.9 | Unspecified persistent mood disorder |
| F41.2 | Mixed anxiety and depressive disorder |
| F53.0 | Postnatal depression |

## Appendix II. Depression prevalence and logistic regression

Table S2. Depression prevalence (%) with confidence interval between years

|  | **Prevalent cases** | **Population** | **Prevalence** | **95% CI** | **Change %** |
| --- | --- | --- | --- | --- | --- |
| 2017 | 124.904 | 5474794 | 2.28 | (2.27; 2.29) | - |
| 2018 | 148.092 | 5159122 | 2.87 | (2.86; 2.88) | 25.82 |
| 2019 | 173.601 | 5567310 | 3.12 | (3.10; 3.13) | 8.63 |
| 2020 | 191.401 | 5684132 | 3.37 | (3.35; 3.38) | 7.99 |
| 2021 | 218.319 | 5710331 | 3.82 | (3.81; 3.84) | 13.54 |
| 2022 | 245.799 | 5773844 | 4.26 | (4.24; 4.27) | 11.35 |

Table S3. Logistic regression results

|  | OR | 95% CI | *p*-value |
| --- | --- | --- | --- |
| Sex |  |  |  |
| Men | 1.06 | (1.04; 1.09) | <0,001 |
| Age |  |  |  |
| 0-15 | 1.98 | (1.85; 2.13) | <0,001 |
| 16-24 | 1.27 | (1.21; 1.34) | <0,001 |
| 25-34 | 1.12 | (1.08; 1.18) | <0,001 |
| 35-44 | 1.02 | (0.98; 1.06) | 0424 |
| 55-64 | 0.99 | (0.96; 1.03) | 0688 |
| 65-74 | 0.81 | (0.78; 0.85) | <0,001 |
| 75-84 | 1.00 | (0.96; 1.04) | 0910 |
| +85 | 1.99 | (1.90; 2.08) | <0,001 |
| Recurrent |  |  |  |
| Yes | 1.37 | (1.32; 1.43) | <0,001 |
| Rurality |  |  |  |
| Urban | 0.93 | (0.91; 0.95) | <0,001 |
| Antidepressants | |  |  |
| Yes | 1.54 | (1.50; 1.57) | <0,001 |
| Anxiolytics |  |  |  |
| Yes | 1.06 | (1.03; 1.09) | <0,001 |

*Reference categories: Women, aged 45-54, non-recurrent, rural area, not taking antidepressants or anxiolytics.
